# Carbon and Nitrogen Isotopes from Top Predator Amino Acids Reveal Rapidly Shifting Ocean Biochemistry in the Outer California Current

**DOI:** 10.1371/journal.pone.0110355

**Published:** 2014-10-17

**Authors:** Rocio I. Ruiz-Cooley, Paul L. Koch, Paul C. Fiedler, Matthew D. McCarthy

**Affiliations:** 1 Ocean Sciences Department, University of California Santa Cruz, Santa Cruz, California, United States of America; 2 Earth and Planetary Sciences Department, University of California Santa Cruz, Santa Cruz, California, United States of America; 3 Southwest Fisheries Science Center, National Marine Fisheries Service, National Oceanic and Atmospheric Administration, La Jolla, California, United States of America; University of California, Merced, United States of America

## Abstract

Climatic variation alters biochemical and ecological processes, but it is difficult both to quantify the magnitude of such changes, and to differentiate long-term shifts from inter-annual variability. Here, we simultaneously quantify decade-scale isotopic variability at the lowest and highest trophic positions in the offshore California Current System (CCS) by measuring δ^15^N and δ^13^C values of amino acids in a top predator, the sperm whale (*Physeter macrocephalus*). Using a time series of skin tissue samples as a biological archive, isotopic records from individual amino acids (AAs) can reveal the proximate factors driving a temporal decline we observed in bulk isotope values (a decline of ≥1 ‰) by decoupling changes in primary producer isotope values from those linked to the trophic position of this toothed whale. A continuous decline in baseline (i.e., primary producer) δ^15^N and δ^13^C values was observed from 1993 to 2005 (a decrease of ∼4‰ for δ^15^N source-AAs and 3‰ for δ^13^C essential-AAs), while the trophic position of whales was variable over time and it did not exhibit directional trends. The baseline δ^15^N and δ^13^C shifts suggest rapid ongoing changes in the carbon and nitrogen biogeochemical cycling in the offshore CCS, potentially occurring at faster rates than long-term shifts observed elsewhere in the Pacific. While the mechanisms forcing these biogeochemical shifts remain to be determined, our data suggest possible links to natural climate variability, and also corresponding shifts in surface nutrient availability. Our study demonstrates that isotopic analysis of individual amino acids from a top marine mammal predator can be a powerful new approach to reconstructing temporal variation in both biochemical cycling and trophic structure.

## Introduction

The California Current System (CCS) contains one of the five major coastal upwelling zones in the world’s oceans, and hosts a great diversity and abundance of marine life [1]. The oceanographic state of this large ecosystem is dynamic. Natural climate variation and anthropogenic stressors alter biochemical cycling, food web dynamics, and the fitness of species [1]–[3]. Known interannual and decadal changes are related both to the El Niño-Southern Oscillation (ENSO) and to basin-scale processes associated with the Pacific Decadal Oscillation (PDO) [1].The latter, is an index of interannual sea surface temperature (SST) variability in the North Pacific, that is related to physical and biochemical variations and influences community changes in plankton, fish and other taxa [4], [5]. In addition to this natural variability, humans have perturbed climate by increasing atmospheric CO_2_ concentrations, which have increased ocean temperatures, water column stratification, hypoxia, and water column anoxia and have decreased surface ocean pH [6], [7]. These environmental factors may negatively impact populations of species, increasing mortality and decreasing reproductive success due to habitat compression and metabolic constraints [8]. Other anthropogenic pressures, such as intensive fisheries and the past whaling industry (which principally targeted sperm whales, *Physeter macrocephalus*) might have triggered top-down effects. Given the lack of detailed proxy records to trace simultaneously biochemical baselines and length of food webs, assessing the extent to which biogeochemical cycling and community structure in pelagic ecosystems have changed over the past century is difficult, as is attributing change to natural cycles versus anthropogenic disturbances.

The isotopic values of marine primary producers are sensitive to environmental variation, such as change in temperature, and CO_2_ or nitrate concentrations, as well as biological differences such as physiology and growth rate [9]–[11]. Hence, the carbon and nitrogen isotope values (δ^13^C and δ^15^N values, respectively) of primary producers, also known as “baseline isotope values”, vary in space and time as a function of these fundamental ecosystem properties [12]. Baseline isotope values are then integrated into consumers’ tissues through diet, typically with metabolic fractionation leading to enrichment in the heavier isotope (especially ^15^N) in consumers [13], [14]. Therefore, isotopic values of marine consumers could be used to reconstruct changes in diet and/or ecosystem biogeochemistry. The δ^13^C and δ^15^N values from a resident animal can potentially provide an integrated record of the biogeochemical characteristics of its habitat, as well as its trophic position [15]. However, because multiple factors influence the bulk δ^13^C and δ^ 15^N values ultimately recorded in consumer tissues, it is often difficult to disentangle the effects of changing trophic position from shifts in baseline values.

Studies in different ocean basins have shown that bulk tissue δ^13^C or δ^15^N values have declined over the last century, but interpretations of these trends have varied widely [16]. For example, declining bulk tissue δ^ 15^N values are sometimes attributed to a drop in consumer trophic level [17], [18] or to baseline shifts due to either changes in foraging zone or biogeochemical cycles [16]. In particular, two recent studies in the Pacific have revealed pervasive declines in δ^ 15^N values in the offshore Central Pacific [18] and North Pacific Subtropical Gyre (NPSG) [19], but offered diametrically opposing interpretations as to underlying mechanism. In the highly productive CCS, despite accumulating evidence for oceanographic changes since the 1950s [2], isotopic data from plankton species have been contradictory. Bulk δ ^15^N values from three zooplakton species have exhibited no long-term trends, whereas data for a specialized zooplankton feeder decreased by approximately 3‰ [20,21]. Declines in δ^13^C values over the 20^th^ century are expected due to the combustion of fossil fuels (i.e., the Suess effect), and have been observed in many records and ecosystems [22]. However, variability in the magnitude and timing of δ^13^C declines has suggested that other factors, such as declining primary productivity, could also contribute in some regions [16]. In the offshore CCS, there are currently no δ^13^C time series for organic or inorganic material.

Isotopic analysis of individual amino acids (AAs) can effectively separate trophic effects from shifts in baseline isotope values [23], [24]. Regardless of an animal’s trophic position, the original δ^15^N and δ^13^C values from primary producers are relatively well preserved within the group of ‘source-AAs’ for nitrogen [25] and the ‘essential-AAs’ for carbon [26]. In contrast, isotopic values from the ‘trophic-AAs’ for nitrogen, and ‘non-essential-AAs’ for carbon, undergo significant metabolic fractionation, and vary in association with a consumer’s diet [23], [24], tissue turnover rates, and possibly metabolism [27]. Hence, isotopic analysis of amino acids from apex marine mammal predators offers a unique opportunity to simultaneously investigate temporal variation at the lowest and highest trophic levels of their food web. Sperm whales are top predators of the mesopelagic ocean. Mark-recapture studies, morphology, and acoustic analysis indicate that female sperm whales forage within the same oceanic region year round [28]. Consequently, they can function as natural biological samplers, broadly integrating biogeochemical information from their home ecosystem. In this study, we use sperm whale skin as a novel biological archive of time series data. Our data combine bulk tissue and AA isotope analysis to examine temporal variation in baseline values (reflecting ecosystem biogeochemistry) and whale trophic position (indicating trophic structure) from offshore waters of the California Current ecosystem.

## Results and Discussion

### Foraging zone of sperm whales sampled in CCS

**Figure 1:**
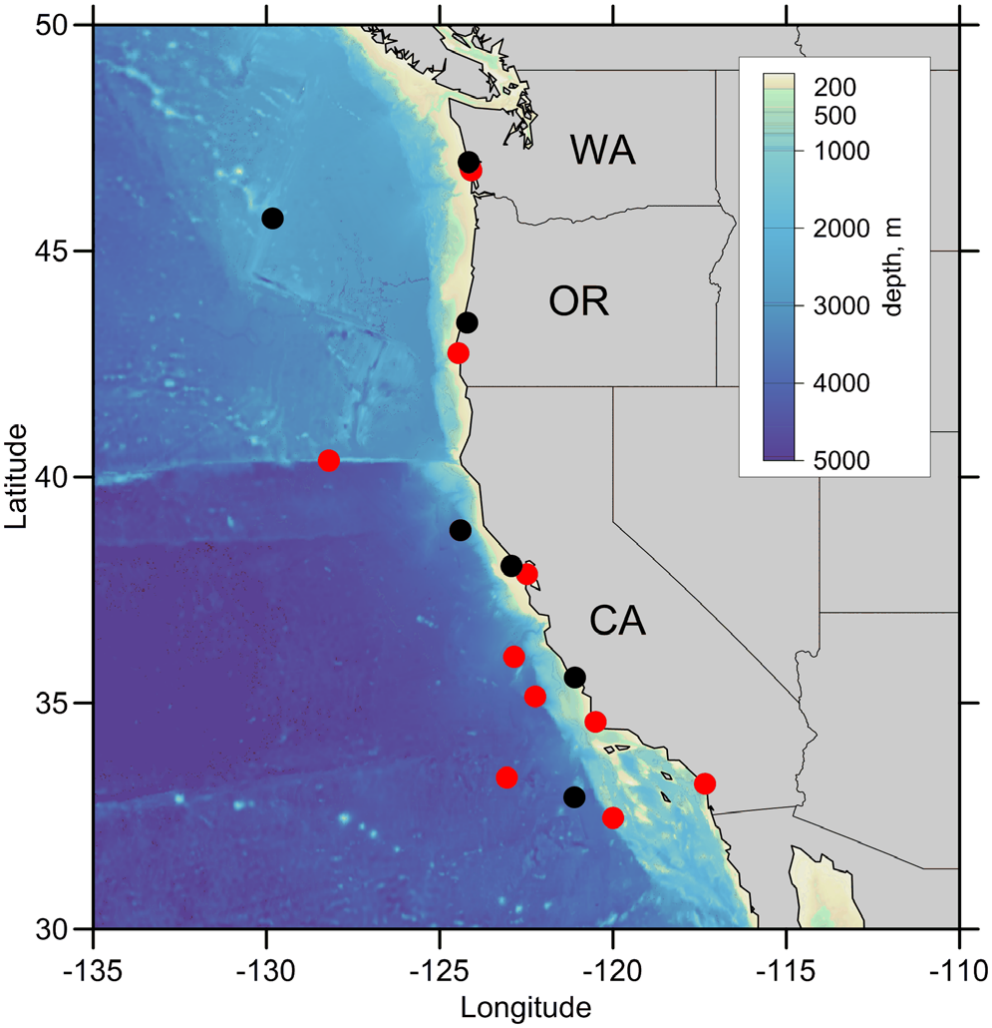
Sperm whales are distributed year-round in offshore deep waters (∼>150 km off the US west coast [29]). Skin samples (○) from free-ranging sperm whales were collected together with skin from stranded individuals. Tissue samples were used for bulk (in black) and amino acid (in red) stable isotope analysis.

In the CCS off the US west coast, sperm whales are found in oceanic waters from California to Washington [29]. Their habitat therefore excludes the coastal upwelling system that exhibits strong latitudinal isotopic gradients [30]. Mitochondrial and nuclear markers reveal that the CCS whales are an independent population and a single genetic stock [31]. Our isotopic data from skin biopsies (Figure 1) indicate that whales fed homogenously within the offshore northern and central CCS. First, the variation in bulk isotope values (n = 18; SD = 1.2‰ for δ^13^C and 1.2‰ for δ^15^N) is similar to the variation observed in other sperm whale populations that are considered to be resident (i.e., Gulf of Mexico and Gulf of California, SD≤0.8‰ for both δ^15^N and δ^13^C [15]; SE Pacific, SD = 3.5‰ for δ^15^N and 0.7‰ for δ^13^C [32]). In addition, the δ^15^N values for phenylalanine (Phe; n = 12; mean (SD)  = 10.9‰ (0.9)) are relatively consistent with expected nitrate and particulate organic matter δ^15^N values from the oceanic northern CCS (∼6 to 10 ‰) [12], and also with published Phe δ^15^N values from muscle of the jumbo squid (*Dosidicus gigas*; potential prey of sperm whales) [33]. Phe δ^15^N values are a proxy for primary producer values [25] as they exhibit only minor ^15^N-enrichment with trophic transfer [23]. In top predators (such as sperm whales), this likely results in slightly higher Phe δ^15^N values versus baseline inorganic N sources. Lastly, because latitudinal trends in the δ^15^N values from predator source-AAs can indicate their geographic residency [24], [33], the lack of any latitudinal variation in Phe δ^15^N values (r ^2^ = 0; n = 12) strongly suggests that the individual sperm whales sampled here were not foraging in different localized regions, but rather foraged over a broad latitudinal range within the northern and central CCS. While the isotopic incorporation rate for extremely large animals like whales is not well known, the thick skin of sperm whales likely integrates information for at least three and possibly more than six months prior to sampling [34]. Our data set encompasses information mainly from the fall and winter, except for the samples collected in 2001 and 2003, which also integrate information from the summer.

### Coupled decadal declines in δ^15^N and δ^13^C values

**Figure 2:**
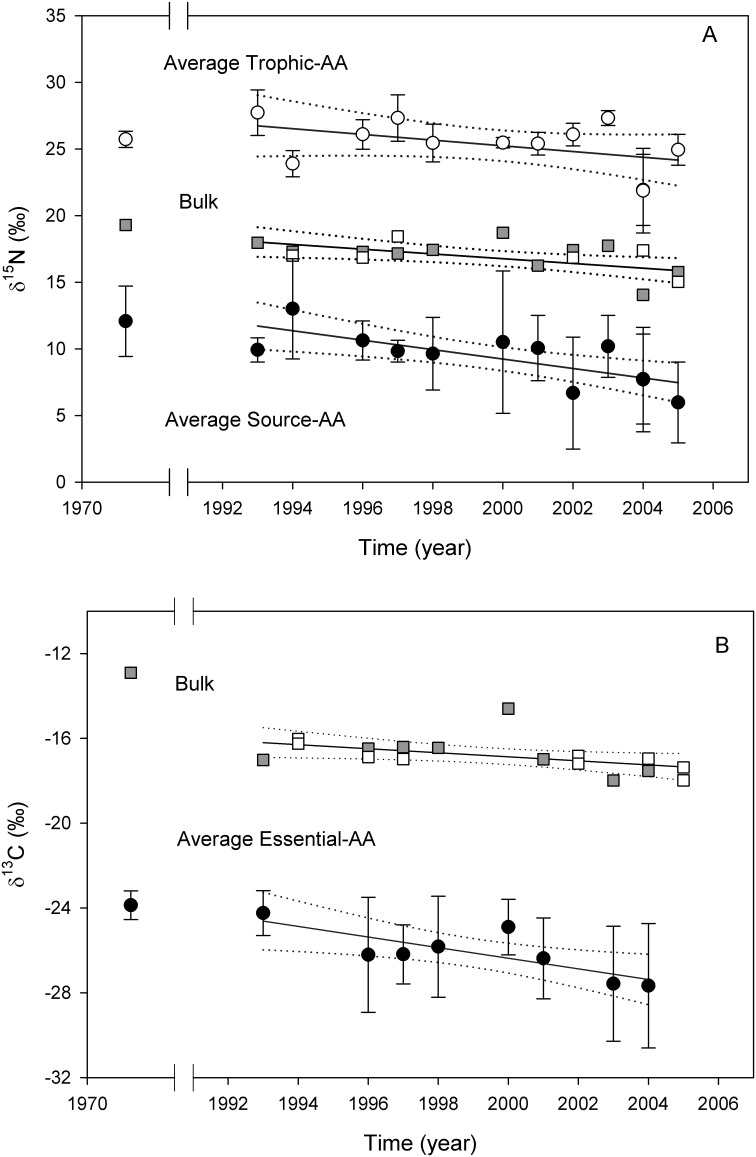
Time series of isotopic data from sperm whale skin. (A) δ^15^N values from bulk skin, average source-AAs and average trophic- AAs (± SD); and (B) δ^13^C values from bulk skin and average essential-AAs (±SD). Bulk isotope data are plotted with a square symbol (□), filled grey squares indicate the samples that were also analyzed for amino acid stable isotope analysis. The corresponding linear regression equations are provided in Table 1, as are the amino acids included within each AA-group.

Bulk δ^13^C and δ^15^N values in whale skin decreased from 1993 to 2005 by 1.1‰ and 1.7‰, respectively. These decreases were statistically significant at the alpha = 0.05 level. Inclusion of a single sample available from 1972 further suggests possible longer-term temporal declines for both δ^13^C and δ^15^N values by ≥4‰ and >3‰, that are also statistically significant (Table 1). Together, these coupled time-series declines in bulk δ^13^C and δ^15^N values suggest coincident biogeochemical or trophic system perturbation (Table 1, Figure 2). In particular, the rate of decrease for bulk δ^15^N values since the 1970’s is at least five times greater than the rate for the long-term δ^15^N decrease recently documented in the central Pacific from proteinaceous corals (2.3‰ in 150 years; annual decrease calculated at 0.015‰) [19], and it is more similar to the rate of change observed for a single zooplankton δ^15^N record from southern California (∼3‰ in 50 years) [21].

**Table 1:** Temporal variation in δ^ 15^N and δ^ 13^C values from the offshore California Current System in sperm whale skin samples.

Time period	Tracer	Linear Regression	n	*r* ^2^	*p*-value	Isotopicshift(‰)	Annualdecrease
1993–2005	δ^15^N						
	Bulk	y = 302–0.143 * year	17	0.25	<0.05	1.7	0.14
	Mean Source-AA	y = 717–0.354 * year	11	0.52	= 0.01	4.2	0.35
	Mean Trophic-AA	y = 311–0.143 * year	11	0.12	>0.05	1.7	
1972–2005	δ^15^N						
	Bulk	y = 218–0.101 * year	18	0.37	<0.05	3.3	0.10
	Mean Source-AA	y = 298–0.145 * year	12	0.39	<0.05	4.7	0.14
	Mean Trophic-AA	y = 88–0.031 * year	12	0.03	>0.05	1.0	
1993–2005	δ^ 13^C						
	Bulk	y = 174–0.095 * year	17	0.24	<0.05	1.1	0.09
	Mean Essential-AA	y = 474–0.250 * year	8	0.62	<0.05	3.0	0.25
1972–2005	δ^13^C						
	Bulk	y = 242–0.129 * year	18	0.67	<0.01	4.2	0.12
	Mean Essential-AA	y = 184–0.105 * year	9	0.58	<0.05	3.4	0.10

For mean calculations: Source-AAs are phenylalanine, glycine, lysine, tyrosine; Trophic-AA: glutamic acid, alanine, isoleucine, leucine, proline; Essential-AA: phenylalanine, valine, leucine. Isotopic shifts were calculated using the corresponding linear regression equations listed in this table. The annual decrease was calculated for shifts that exhibited a *p*-value≤0.05.

To disentangle the factors driving the declines in bulk isotope values, we analyzed individual AA isotope values, focusing on AAs that have been demonstrated to track baseline changes (as noted above, essential AA for δ^13^C values, source AA for δ^15^N values). Linear regression models for average δ^13^C and δ^15^N values from the most accurately measured essential- and source-AAs both exhibited strong negative temporal trends across all the data (i.e. for both 1972 and 1993 to 2005; Table 1, Figure 2), with drops of ≥3‰ and >4‰ respectively indicated by compound-specific isotope data. Residuals for all regressions exhibited a random pattern. In contrast, average δ^15^N values for the trophic-AAs were much variable, resulting in a lower r^2^, but overall they paralleled the source-AA trend (Table 1, Figure 2A). These results are not consistent with any significant drop in sperm whale trophic level as the primary driver of decreases in bulk isotope ratios, and instead strongly implicate coupled changes in baseline δ^15^N and δ^13^C values.

These negative trends in baseline δ^15^N and δ^13^C values might relate to changes in biochemical cycling, rates of primary production, or primary producer species composition. In particular, the decline in average essential-AA δ^13^C values (Figure 2B), which are a direct proxy for primary producers, is far too high to be explained solely by the Seuss effect (∼0.2 ‰ per decade since 1960 [35]), and it also coincides with the decline in average source-AA δ^15^N values. This suggests that the mechanism explaining a drop in primary producer δ^15^N values should be consistent with a concurrent large decline in δ^13^C values. One possiblity, which would represent a direct analogy to changes in other ocean regions, would be a shift towards more oligotrophic conditions for the outer CCS. This explanation would be consistent with coupled declines in both isotopes, linked to decreased primary production and a shift in species composition that is typically associated with warmer and more stratified ocean conditions [36]. Oligotrophy in the world ocean is increasing due to climate shifts [37] and is projected to continue increasing in the North Pacific [38]. Recent isotopic records from deep sea proteinacous corals, for example, provide strong support for such linked trends associated with warming of the NPSG [39]. The nitrogen isotope record from deep sea coral indicate that the long-term declines in baseline δ^15^N values are likely linked to progressive increases in seasonal gyre extent, leading to steady increases in N contribution from diazotrophy [19]. Therefore, an analogous explanation would imply that oceanographic conditions in the offshore CCS region (which have conditions more similar to the open ocean and represent the base of sperm whales’ food web) might have shifted toward more “gyre-like” conditions, driving baseline isotope values toward those more typical of the oligotrophic open ocean.

**Figure 3:**
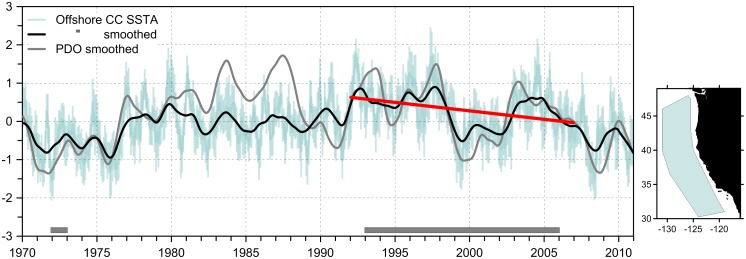
Time series data of sea surface temperature anomaly (SSTA) from the offshore California Current (inset map) and the Pacific Decadal Oscillation (PDO). Monthly SSTA was computed in 0.5-deg fields from the Simple Ocean Data Assimilation version 2.2.4 reanalysis (http://coastwatch.pfeg.noaa.gov/erddap/griddap/hawaii_d90f_20ee_c4cb.html), and then averaged in the offshore area (the plot shows ±1sd). Monthly SSTA (°C) and PDO values were smoothed with a 25-month lowess smooth. The linear fit is for 1992–2006 (red line, slope −0.044°C y-1). Sample periods are indicated along the time axis.

However, to our knowledge, there is currently no evidence for substantially increasing SST and diazotrophy in the CCS itself. Instead, recent analyses suggest largely the opposite: overall, the thermocline weakened and shoaled in the offshore CCS between 1950 and 1993 [7], possibly increasing nutrient availability in the euphotic zone despite increased stratification [40]. Additionally, the offshore CCS has cooled (not heated) since the early 1990s (Figure 3), and this trend is also reflected in the present “cool” PDO regime. Furthermore, the generalization that global warming will universally increase stratification and thus decrease surface nutrient supply has been recently challenged for some regions including the CCS [41]. For example, one recent model projects increases in nitrate supply and productivity in the CCS during the 21st century despite increases in stratification and limited change in wind-driven upwelling [42]. In the southern CCS, coastal surface nutrients have increased possibly linked to a general shoaling of the nutricline [43]. In the Southern California Bight, the most intensively monitored region of the CCS, nutrients in source waters have also increased over the last three decades, but the N:P and Si:N ratios were greatly reduced, possibly shifting phytoplankton species composition and abundance [44]. Whether or not these trends in nutrient dynamics extend to other regions of the CCS is unclear, because the oceanographic state of this ecosystem varies regionally [1], [45].

In particular, shifts in offshore and onshore oceanographic conditions appear to be decoupled. Coastal upwelling has recently increased, as expected for enhanced alongshore winds [46], but has decreased offshore where upwelling is driven by wind-stress curl [47]. Since 1997, trends in satellite chlorophyll estimates, an index of phytoplankton biomass, have been positive in coastal upwelling waters but tend to be zero or negative in offshore waters [48]. Together, this current evidence indicates cooling, but not increases in productivity, in the offshore CCS concurrent with the observed 1993–2006 trends in sperm whale δ^15^N and δ^13^C values. Lower temperatures increase the solubility of CO_2_ and change the fractionation associated with carbon fixation, often resulting in lower phytoplankton δ^13^C values [49]; lower temperatures might have also changed phytoplankton growth and species composition. If surface nitrate also increased along the outer CCS region sampled by these whales, then the degree of nitrate utilization by primary producers (and so their δ^15^N values) could have also changed, since phytoplankton preferentially assimilate ^14^NO_3^−^_ [50]. In general, proportional nitrate utilization is lower where surface NO_3^−^_ concentrations are higher [50]. Therefore, lower NO_3^−^_ utilization during seasonal upwelling might also be expected to depress the δ^15^N values of primary producers, propagating the ^15^N-depleted signal into food webs during their most productive periods. At present, there simply are not enough detailed data on nutrient concentrations and other oceanographic factors in the outer CCS to deduce a mechanism. However, the observed declining baseline values revealed by sperm whales do indicate a recently progressive shift in primary producer dynamics, likely associated with changes in SST, average state of surface nutrients and/or primary production.

### Implications of Rapid Change for offshore CCS Biogeochemistry

Although our time series data are limited for both elements, the compound-specific AA data identify a parallel decline in both baseline δ^15^N and δ^13^C values in the outer CCS from 1992 to 2005, likely indicative of major recent shifts in biochemical cycling. At the same time, however, the overall similarity in whale trophic position signifies that the broad trophic structure is realtively unaffected. We note that in comparison with the recent deep sea coral data from the gyre offshore of this region [19], our data suggest that both the rate and scale of biochemical change on the CCS margin may be far greater than in the open Pacific Ocean. The coral record from the NPSG indicates a fairly steady δ^15^N annual decrease of ∼0.015‰ over the last 150 years with a total drop of 2.3 ‰ in exported primary production δ^15^N values over that period. In contrast, our molecular-level proxies for δ^15^N values at the base of the food chain (the source AAs) indicate more rapid annual declines of 0.35 ‰ since the 1990’s. The independent molecular proxies for primary production δ^13^C values (the essential AAs) indicate relatively similar declines.

Together with the CCS observations discussed above, the contrast with the NPSG coral data (while not directly comparable in terms of time scale), suggests that despite the fact that baseline δ^15^N declines are observed in both data sets, different biogeochemical mechanisms may underlie the changes in these very different oceanographic regions. Climate variability likely affects the biochemistry of ecosystems differently depending on the oceanographic properties, microbial and phytoplankton communities, and species assemblages. In the eastern Pacific Ocean, the structure of the pycnocline varies strongly among the known biogeochemical provinces [51]. This likely influences geographic variation in surface nutrient availability, and therefore stable isotope ratios in POM, primary producers [12] and consumers [14]. Temporal trends in pycnocline depth, SST, stratification, and mixed layer depths also differ between these biogeochemical provinces [40]. For example, while SST decreased overall since 1958 in many parts of the California Current, SST increased in the easternmost southern subtropical gyre and equatorial Pacific [40]. Ultimately, more detailed data that couple integrated measures of ecosystem baseline with oceanographic state will be required to understand the substantial biogeochemical changes our data indicate.

Our work highlights that detailed time-series of biochemical baseline and trophic structure records among different ecosystems will be crucial to identify rapid ecosystem shifts in response to climate change. In particular, in the face of uncertain coupling of natural and anthropogenic climate forcing, understanding the timing, extent and especially the mechanistic basis for baseline shifts now represents an urgent challenge. However, despite many efforts to unravel the linkage and feedback controls between the carbon and nitrogen cycles, and the effect of their variability on primary production and food-web dynamics, they are still not well understood. This study has demonstrated the great potential in coupling molecular isotopic tools with the unique bioarchive of sperm whales (or other top predators), as sentinels of offshore ecosystems. This may allow, for the first time, decoding of the factors that underlie temporal trends in bulk isotopic records, while simultaneosly monitoring changes at both the highest and lowest trophic levels. We suggest that integrating this approach with detailed oceanographic data will be a major new tool to identify the effects of natural climate variability versus anthropogenic global warming on ecosystem biochemistry and primary production. Elucidating such patterns from this and other ocean margin regions, in particular their relationships with oceanographic and climatic variations and shifts in primary production, will be an essential part of the critical task of predicting future trends in both ecosystem biochemistry and trophic dynamics.

## Material and Methods

A total of 18 skin samples (Figure 1) were analyzed for bulk stable isotope analysis. Skin tissue samples with enough material (3.5 mg) were selected for CSIA-AA. Data from 12 samples were obtained for individual AA δ^15^N values, and 9 samples for δ^13^C values. The Southwest Fisheries Science Center/Pacific Islands Fisheries Science Center Institutional Animal Care and Use Committee (IACUC) approved the original animal work that produced the samples. Sex was determined genetically using qPCR sexing assay by the PRD-Genetic Lab at NOAA [52]. These samples consisted of 5 females, 2 males and 2 unidentified individuals possibly corresponding to females or juvenile males. Large adult males were not included. Bulk isotope values were analyzed by continuous flow isotope ratio mass spectrometry (IRMS; Thermo Finnigan) and standardized relative to Vienna-Pee Belemnite (V-PDB) for carbon and atmospheric N_2_ for nitrogen. Results are expressed in part per thousand (‰) and standard notation: δ^H^X = [(R_sample_/R_standard_)−1]×1000, where H is the mass number of the heavy isotope, X is either C or N, and R_sample_ and R_standard_ are the ratio of ^13^C/^12^C or ^15^N/^14^N in the sample and standard, respectively.

We hydrolyzed and prepared approximately 3.5 mg of skin as well as a control (Cyanno; bacteria tissue) [53] to quantify δ^15^N values from source- and trophic-AAs and δ^13^C values from essential- and non-essential-AAs. All derivatives were injected with an AA control, N-leucine, to verify accuracy during each run, and analyzed via gas chromatography-IRMS to obtain δ^15^N and δ^13^C values from individual AAs. Each sample was run 3–4 times to maximize accuracy among chromatograms. The associated analytical error among replicates was <1.0 ‰. For all samples, δ^15^N values were obtained from a total of four source-AAs (phenylalanine, glycine, lysine, tyrosine), and five trophic-AAs (glutamic acid, alanine, isoleucine, leucine, proline) (Figure S1A). For δ^13^C values, the essential-AAs that we consistently determined were phenylalanine, valine and leucine, and the non-essential-AA were alanine, proline, aspatic acid, glutamic acid and tyrosine (Figure S1B).

The relative pattern of AA δ^15^N and δ^13^C values was highly consistent with past work from other organisms and tissues [23], [25], [54]. We grouped data as source- or trophic-AAs for δ^15^N values, and essential- or non-essential-AAs for δ^13^C values to increase power in the analysis and evaluate temporal variation. We calculated average values for each AA group and they are reported in Table S1. Regression analyses were conducted to evaluate linear relationship between time and each isotopic tracer for both bulk and individual-AA δ^15^N and δ^13^C values (Table 1).

There was a weak correlation between average source-AA and trophic-AA (r^2^ = 0.13; p = 0.67), indicating that trophic-AA δ^15^N values could not be predicted by the variability in source-AAs, and vice versa. However, the correlation between average essential-AA and non-essential-AA δ^13^C values was moderate (r^2^ = 0.63, p = 0.06). Since the controls on isotopic patterns for non-essential-AA δ^13^C values are complex and dependent on diet quality and quantity, including *de novo* synthesis and routing of AAs from diet-to-tissue, this group was not considered in the linear regression analysis.

## Supporting Information

Figure S1 **Stable isotope values of individual amino acids (AAs) in skin samples of sperm whales (** ***Physeter macrocephalus*** **).** (A) Four δ^15^N Source-AAs: phenylalanine (phe), glycine (gly), lysine (lys), tyrosine (tyr), and five Trophic-AAs: glutamic acid (glx), alanine (ala), isoleucine (ile), leucine (leu), proline (Pro); and (B) Three δ^13^C essential-AAs: phe, leu, and valine (val).
(TIF)

Table S1 **Average values and one standard deviations (SD) were calculated for Source-AAs (phenylalanine, glycine, lysine, tyrosine), Trophic-AAs (glutamic acid, alanine, isoleucine, leucine, proline) and Essential-AAs (phenylalanine, valine, leucine).**
(DOCX)
