# Supplementary figures and images for: Carbon and Nitrogen Isotopes from Top Predator Amino Acids Reveal Rapidly Shifting Ocean Biochemistry in the Outer California Current
Source: PLoS One. 2014 Oct 17;9(10):e110355. doi: 10.1371/journal.pone.0110355 (PMC4201512; doi:10.1371/journal.pone.0110355)

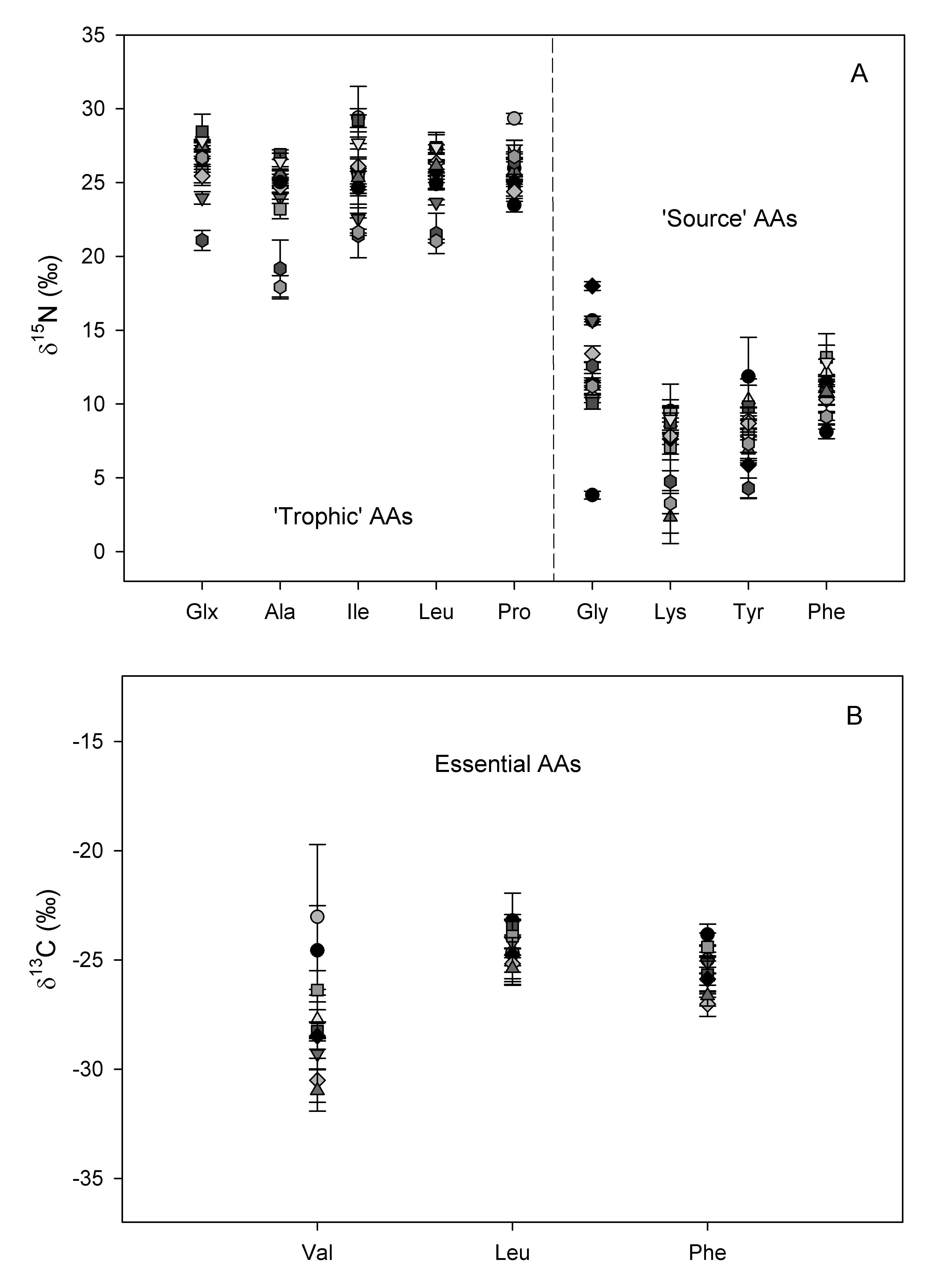

Supplement: Figure S1 — Stable isotope values of individual amino acids (AAs) in skin samples of sperm whales ( Physeter macrocephalus ). (A) Four δ15N Source-AAs: phenylalanine (phe), glycine (gly), lysine (lys), tyrosine (tyr), and five Trophic-AAs: glutamic acid (glx), alanine (ala), isoleucine (ile), leucine (leu), proline (Pro); and (B) Three δ13C essential-AAs: phe, leu, and valine (val). (TIF) [file pone.0110355.s001.tif]
