# Supplementary material for: Carbon and Nitrogen Isotopes from Top Predator Amino Acids Reveal Rapidly Shifting Ocean Biochemistry in the Outer California Current
Source: PLoS One. 2014 Oct 17;9(10):e110355. doi: 10.1371/journal.pone.0110355 (PMC4201512; doi:10.1371/journal.pone.0110355)
Supplement: Table S1 — Average values and one standard deviations (SD) were calculated for Source-AAs (phenylalanine, glycine, lysine, tyrosine), Trophic-AAs (glutamic acid, alanine, isoleucine, leucine, proline) and Essential-AAs (phenylalanine, valine, leucine). (DOCX) [file pone.0110355.s002.docx]

| Year | 1972 | 1993 | 1994 | 1996 | 1997 | 1998 | 2000 | 2001 | 2002 | 2003 | 2004 | 2005 |
| --- | --- | --- | --- | --- | --- | --- | --- | --- | --- | --- | --- | --- |
| δ^15^N |  |  |  |  |  |  |  |  |  |  |  |  |
| Source | 12.1 | 9.9 | 13.0 | 10.6 | 9.8 | 9.6 | 10.5 | 10.1 | 6.7 | 10.2 | 7.7 | 6.0 |
| SD | 2.6 | 0.9 | 3.7 | 1.5 | 0.8 | 2.7 | 5.3 | 2.4 | 4.2 | 2.3 | 3.9 | 3.0 |
| Trophic | 25.7 | 27.7 | 23.9 | 26.1 | 27.3 | 25.4 | 25.5 | 25.4 | 26.1 | 27.3 | 21.9 | 24.9 |
| SD | 0.6 | 1.7 | 1.0 | 1.1 | 1.7 | 1.4 | 0.4 | 0.8 | 0.8 | 0.5 | 2.7 | 1.1 |
| δ^13^C |  |  |  |  |  |  |  |  |  |  |  |  |
| Essential | -23.9 | -24.2 |  | -26.2 | -26.2 | -25.8 | -24.9 | -26.4 |  | -27.6 | -27.7 |  |
| SD | 0.7 | 1.0 |  | 2.7 | 1.4 | 2.3 | 1.3 | 1.9 |  | 2.7 | 2.9 |  |
